# Cerebrovascular assessment of patients undergoing shoulder surgery in beach chair position using a multiparameter transcranial Doppler approach

**DOI:** 10.1007/s10877-018-0211-7

**Published:** 2018-10-17

**Authors:** Danilo Cardim, Chiara Robba, Basil Matta, Graham Tytherleigh-Strong, Niel Kang, Bernhard Schmidt, Joseph Donnelly, Leanne Calviello, Peter Smielewski, Marek Czosnyka

**Affiliations:** 1 Brain Physics Laboratory, Division of Neurosurgery, Department of Clinical Neurosciences, University of Cambridge, Cambridge, UK; 2 Department of Anesthesiology, Pharmacology & Therapeutics, The University of British Columbia, Vancouver, Canada; 3 Neurosciences Critical Care Unit, Addenbrooke’s Hospital, Cambridge University Hospitals NHS Foundation Trust, Cambridge, UK; 4 Department of Neuroscience, University of Genoa, Genoa, Italy; 5 Department of Anaesthesia and Intensive Care, San Martino Policlinico Hospital, Genoa, Italy; 6 Department of Anaesthesia, Addenbrooke’s Hospital, Cambridge University Hospitals NHS Foundation Trust, Cambridge, UK; 7 Department of Trauma and Orthopaedics, Addenbrooke’s Hospital, Cambridge University Hospitals NHS Foundation Trust, Cambridge, UK; 8 Department of Neurology, University Hospital Chemnitz, Chemnitz, Germany; 9 Department of Anaesthesiology, University of Auckland, Auckland, New Zealand; 10 Institute of Electronic Systems, Warsaw University of Technology, Warsaw, Poland; 11 Vancouver General Hospital, 899 W 12th Ave, Room 2469, Vancouver, V5Z 1M9 Canada

**Keywords:** Beach chair position, Transcranial Doppler, Non-invasive intracranial pressure, Cerebral autoregulation

## Abstract

Although the beach-chair position (BCP) is widely used during shoulder surgery, it has been reported to associate with a reduction in cerebral blood flow, oxygenation, and risk of brain ischaemia. We assessed cerebral haemodynamics using a multiparameter transcranial Doppler-derived approach in patients undergoing shoulder surgery. 23 anaesthetised patients (propofol (2 mg/kg)) without history of neurologic pathology undergoing elective shoulder surgery were included. Arterial blood pressure (ABP, monitored with a finger-cuff plethysmograph calibrated at the auditory meatus level) and cerebral blood flow velocity (FV, monitored in the middle cerebral artery) were recorded in supine and in BCP. All subjects underwent interscalene block ipsilateral to the side of FV measurement. We evaluated non-invasive intracranial pressure (nICP) and cerebral perfusion pressure (nCPP) calculated with a black-box mathematical model; critical closing pressure (CrCP); diastolic closing margin (DCM—pressure reserve available to avoid diastolic flow cessation); cerebral autoregulation index (Mxa); pulsatility index (PI). Significant changes occured for DCM [mean decrease of 6.43 mm Hg (p = 0.01)] and PI [mean increase of 0.11 (p = 0.05)]. ABP, FV, nICP, nCPP and CrCP showed a decreasing trend. Cerebral autoregulation was dysfunctional (Mxa > 0.3) and PI deviated from normal ranges (PI > 0.8) in both phases. ABP and nCPP values were low (< 60 mm Hg) in both phases. Changes between phases did not result in CrCP reaching diastolic ABP, therefore DCM did not reach critical values (≤ 0 mm Hg). BCP resulted in significant cerebral haemodynamic changes. If left untreated, reduction in cerebral blood flow may result in brain ischaemia and post-operative neurologic deficit.

## Introduction

The beach chair position (BCP) offers several advantages in shoulder surgery, such as a better intra-articular visualization and less risk of neurovascular trauma in comparison to surgery performed in the lateral decubitus position [1]. However, BCP may provoke hypotension in the presence of general anaesthesia, which alters cardiovascular reflex responses [2, 3].

There are published reports of rare, but devastating, neurologic injuries occurring in patients in the beach chair position. These include stroke, spinal cord ischaemia, and transient visual loss [4–11]. The incidence of neurologic complications after shoulder surgery in the beach chair position remains unknown, but a survey of the American Shoulder and Elbow Surgeons (including 32% of responders) indicated that major stroke occurs in 0.0004% of such patients [7].

Because of arterial blood pressure (ABP) hypotension and the gravitational effects of positioning the head above the level of the heart, such neurologic complications reported after shoulder surgery in the beach chair position may be the result of cerebral autoregulation failure, inadequate cerebral perfusion, and cerebral ischaemia. By assessing cerebral haemodynamics in patients undergoing surgery in the beach chair position, we aim to clarify whether these issues occur in this scenario.

Transcranial Doppler (TCD) ultrasonography allows continuous and non-invasive assessment of cerebral haemodynamics by monitoring cerebral blood flow velocity (FV). From TCD signal processing, several parameters have been developed and applied in neurocritical care settings, which include cerebral autoregulation indices (CA), critical closing pressure (CrCP) of the cerebral circulation, non-invasive estimations of intracranial pressure (nICP) and cerebral perfusion pressure (nCPP). There are few published reports on the use of TCD in beach chair position for the assessment of cerebral haemodynamics, but unfortunately, most simply use mean FV as the main measure [12, 13]. We advocate for a complete assessment of cerebral haemodynamics including not only pre-existing TCD parameters, like the components of cerebral blood flow velocity (systolic, diastolic, mean), but also the interactions between various parameters derived from mathematical models describing cerebral blood flow circulation.

We hypothesised that BCP may incidentally cause subtle deterioration of cerebral haemodynamics; this can be monitored by transcranial Doppler parameters during shoulder surgery under general anaesthesia. Therefore, the objective of this study is to assess cerebral haemodynamics using a multiparameter TCD-derived approach in patients undergoing shoulder surgery who were positioned from supine to beach chair position.

## Methods

### Patient population

Thirty-three patients with no history of neurological disease having elective shoulder surgery in the beach chair position were enrolled in this study. Patients were admitted to the Department of Trauma and Orthopaedics at Addenbrooke’s Hospital, Cambridge, UK, between March 2016 to 2017. The experimental protocol and informed consent were approved by the Institutional Review Board (REC 16/LO/0350). Informed consent was obtained from all individual participants included in the study.

On arrival to the surgical suite, standard monitoring was applied, including pulse-oximetry, electrocardiography, and non-invasive arterial blood pressure in the arm. The patients did not receive anxiolytic medication prior to induction of anaesthesia. After pre-oxygenation, general anaesthesia was induced with intravenous propofol (2 mg/kg). To facilitate endotracheal intubation, cisatracurium besilate (0.15 mg/kg) was administered intravenously, and then the lungs were ventilated with a mixture of oxygen/air and sevoflurane (MAC of 0.8–1.0) to maintain an end-tidal carbon dioxide (ETCO_2_) of 4.6–5.5 kPa.

Ultrasound guided interscalene block using an in-plane approach was performed to provide intra- and post-operative analgesia. Bupivacaine 0.25–0.5% (15–20 ml) were injected around the superior and middle trunks of the brachial plexus, between the anterior and middle scalene muscles. The plexus regional technique was performed after general anaesthesia. A catheter was not placed for post-operative analgesia in any cases.

For these patients, we administered crystalloids before and during anaesthesia induction or small boluses of metaraminol to improve mean ABP when it presented below 65 mm Hg. The dose and choice of the strategy to maintain an adequate blood pressure depended on many factors, including the patient’s signalment, physical condition, and the length and type of the procedure.

Potential neurological complications were assessed through clinical neurological examination of the patient at the end of the procedure and post-operative period in the recovery room, including the assessment of focal or global deficit and pupil characteristics.

### Monitoring and data analysis

ABP was non-invasively measured for clinical purposes with a cuff via an oscillometric method from the arm (ABP_ARM_). Any clinical interventions to control ABP were performed considering measurements obtained with this method. Additionally, ABP was monitored over time non-invasively using a finger-cuff (Finometer Pro®, Finapres Medical Systems, Amsterdam, The Netherlands) calibrated at the auditory meatus level (ABP_FINGER_) strictly for research purposes. Cerebral haemodynamic parameters were calculated using ABP_FINGER_. Cerebral blood flow velocity was assessed using transcranial Doppler (Digi-Lite™, RIMED, Rihanna, Israel) in the middle cerebral artery ipsilateral to the surgerical side. The monitoring sessions were performed by an experienced operator (DC) and the TCD probe was hand-held in place during the entire recording. The use of a headset for probe support was unfeasible due to technical difficulties to accommodate such a structure in the configuration of the surgical chair’s headrest. Only ABP_FINGER_ and FV signals were recorded simultaneously.

ABP_FINGER_ and FV monitoring were performed in two different periods: in supine position, immediately after induction of anaesthesia (phase A); and before commencement of surgery with the patient in beach chair position (phase B). Beach chair position was achieved by tilting the table at an automated smooth pace to an angle range of 45–90° adjusted for surgical exposure on an individual basis (around 30 s). The measurements during phase B started once the surgical team had the patient in an optimal and stable sitting position, and the onset time varied (generally being close to 1 min). ABP_ARM_ was collected retrospectively from the hospital’s electronic records during the study’s analysis phase. Pre-operative (in the anaesthetic room, before anaesthesia induction) and operative values (in the operation room, with the patient in beach chair position) for ABP_ARM_ were collected.

All parameters were calculated and averaged for both phases. Raw signals were digitized using an analog–digital converter (DT 2814, Marlborough, California, United States of America) sampled at a frequency of 100 Hz and recorded with ICM+® (Cambridge Enterprise, http://www.neurosurg.cam.ac.uk/icmplus/). The recorded signals were subjected to manual artefact removal and analysed with ICM+®. All calculations were performed over a 10 s sliding window.

### Multiparameter TCD-derived cerebral haemodynamic assessment

#### Non-invasive estimation of ICP and CPP

The nICP assessment was performed using a ‘black-box’ mathematical model, published and described thoroughly [14]. In this model, the intracranial compartment is considered a black-box (BB) system, with ICP being a system response to the incoming signal ABP [14]. This mathematical model originates from system analysis, which provides a method to describe the transmission characteristics, with input and output signals. The intracranial compartment is indirectly described by a transfer function [15, 16] which connects the assumed input signal, ABP, with the output signal, ICP. Two linear models are first established to depict the relationship between ABP and ICP and that between ABP and FV, yielding two coefficients, *f* and *w*, respectively. By applying this linear mapping function, non-invasive ICP estimation can be performed by first estimating *f* from the coefficient *w* obtained from ABP and FV (TCD characteristics, obtained from datasets of reference patients with raised ICP following traumatic brain injury (TBI) [14]). An estimate of ICP can then be derived from ABP using the calculated *f*. The output data provides a continuous full waveform of nICP (in mm Hg) and constant relationship between FV and ABP. A recent prospective study in TBI patients assessed the model’s 95% limits of accuracy for ICP estimation as ± 10 mm Hg [17]. nICP estimation was performed using a plugin developed and available for the ICM + software® platform. The non-invasive cerebral perfusion pressure measurement was calculated as the difference between mean ABP and nICP (nCPP = ABP–nICP).

#### Cerebral autoregulation

Cerebral autoregulation describes the intrinsic ability of the cerebral vasculature to maintain a stable cerebral blood flow over a wide range of arterial blood pressures. Continuous indices of cerebral autoregulation can be calculated from spontaneous fluctuations of ABP and FV. In this work, the correlation coefficient between ABP and FV, termed Mxa (mean flow index), was calculated [18]. Mxa close to + 1 denotes that slow fluctuations in ABP produce synchronized slow changes in FV indicating defective cerebral autoregulation. Based on previous studies considering TBI patients, negative values or values less than 0.3 indicate intact CA, while positive values above 0.3 indicate failure of CA [19, 20].

#### Pulsatility index

The pulsatility index describes quantitative and qualitative changes in the morphology of the TCD waveform resulting from alterations in CPP. The PI represents the relationship between the difference of FV_s_ (systolic flow velocity) and FV_d_ (diastolic flow velocity) divided by FV_m_ (mean flow velocity). PI is a multifactorial parameter and has been reported as inversely proportional to mean CPP, directly proportional to pulse amplitude of ABP, and non-linearly proportional to the compliance of the cerebral arterial bed, cerebrovascular resistance and heart rate [21].

#### Critical closing pressure

The critical closing pressure of the cerebral circulation denotes a threshold of ABP below which the blood pressure in the brain vessels is inadequate to prevent the collapse and cessation of blood flow (closing pressure) [22]. CrCP can be represented as the sum of ICP and vascular wall tension [22, 23], and given this association with the vasomotor tone of the brain microvasculature, it may explain cerebral perfusion in different pathologies [22, 24–27].

Derived from CrCP and diastolic ABP (ABP_d_), the diastolic closing margin (DCM = ABP_d_–CrCP) of the brain microvasculature can be obtained. Previous works have demonstrated that ABP_d_ below CrCP correlates with loss of measurable FV during diastole [28], which is associated with an increase in CBF pressure passivity in patients following TBI. CrCP was calculated as described in Varsos et al. [27].

### Statistical analysis

Statistical analysis was conducted with R Studio software (R version 3.3.1). Data were tested for normal distribution using the Shapiro–Wilk test and are presented as median and interquartile range (IQR). All parameters assessed were non-parametric in nature. Statistical measurements were performed with the Wilcoxon rank sum test with continuity correction and the Wilcoxon signed rank test for paired comparisons. In addition, we employed a Spearman correlation coefficient matrix to assess correlations between the various parameters considering differences from supine to beach chair position [delta correlations (∆)]. Delta differences between parameters are presented as mean and percentage change. The statistical significance level for all tests was set at *p* < 0.05.

#### Sample size calculation

A sample size calculation for this study was performed using power analysis based on the proportional power calculation for a binomial distribution (power analysis for one proportion), in which a power of 80%, significance level of 5%, and medium Cohen’s “d” effect size (d = 0.5) were considered. This analysis yielded a sample size of 32 individuals for detecting the specified effect. Based on the number of cases in the area admitted to our Department of Trauma and Orthopaedics between March 2016 and 2017, we could recruit 33 eligible patients during the study period.

## Results

The study was completed in 23 patients: 4 patients could not be included due to absence of an insonation window for TCD; 6 patients due to poor recordings of either ABP or FV. The mean age was 50.83 years [range 18–81 years; 18 males (78%)]. The mean monitoring time was 5.40 ± 1.60 (95% confidence interval: 5.07–7.27) min for phase A and 5.30 ± 1.50 (95% confidence interval: 4.65–6.67) minutes for phase B. Table 1 presents demographics, medical, and surgical characteristics of patients undergoing shoulder surgery. Table 2 presents results for all physiological and cerebral haemodynamic parameters assessed in phase A (supine) and phase B (beach chair position). Table 3 includes the respective mean delta changes expressed in percentage between phases A and B, and the correlation coefficients of changes in FV and ABP with changes in other parameters evaluated.

**Table 1:** Patients’ demographic characteristics, medical history, and type of surgical procedures performed

Characteristics	
Age (years), range (min, max)	59 ± 20 (18–81)
Sex, male/female, n (%)	18 (78%)/5 (22%)
BMI (kg/m^2^)	29 ± 4

Medical history	
Hypertension	30%
Diabetes mellitus	13%
Hypercholesterolemia	9%
Myocardial infarction	4%
Obesity	4%
Depression	22%
Asthma	13%
Sleep apnoea	4%
Thyroid nodule	4%
Adrenal mass	4%
Factor XI haemophilia	4%
Gastroesophageal reflux disease	4%

Procedure	
Arthroscopic subacromial decompression	26%
Arthroscopic cuff repair	22%
Shoulder replacement	17%
Arthroscopic acromioclavicular joint	17%
Shoulder stabilization	13%
Arthroscopic shoulder capsular release	4%

*BMI* body-mass index, *Min* minimum age, *Max* maximum age

**Table 2:** Physiological and cerebral haemodynamic parameters assessed in phases A and B

	A	B	*p*-value
ABP_FINGER_	61.71 (52.41–69.30)	48.53 (37.64–66.35)	0.21
FV	40.86 (33.72–50.77)	43.01 (32.06–51.66)	0.27
nICP	4.52 (2.07–9.57)	2.14 (− 0.18 to 9.14)	0.66
nCPP	57.66 (49.54–63.92)	43.79 (37.46–57.66)	0.14
Mxa	0.32 (0.01–0.78)	0.47 (0.20–0.62)	0.94
PI	1.00 (0.85–1.14)	1.03 (0.90–1.34)	0.048
CrCP	31.65 (22.18–37.94)	25.33 (13.15–41.95)	0.60
DCM	18.08 (11.23–23.88)	10.44 (7.78–15.71)	0.01
ETCO_2_	5.30 (4.90–5.40)	5.20 (4.80–5.35)	0.17

ABP_FINGER_ (mm Hg), arterial blood pressure monitored at the auditory meatus level; FV (cm/s), cerebral blood flow velocity; nICP (mm Hg), non-invasive intracranial pressure; nCPP (mm Hg), non-invasive cerebral perfusion pressure; Mxa, cerebral autoregulation index; PI, pulsatility index; CrCP (mm Hg), critical closing pressure; DCM (mm Hg), diastolic closing margin; ETCO_2_ (kPa), end-tidal carbon dioxide concentration; Phase A, supine position; Phase B, beach chair position

**Table 3:** Mean delta changes and correlation coefficients between FV and ABP considering delta changes

	∆ (%)	R with ∆ABP	p-value	R with ∆FV	*p*-value
ABP_FINGER_	− 14	–	–	0.48	0.02
FV	− 6	0.48	0.02	–	–
nICP	− 29	0.61	0.002	0.33	0.12
nCPP	− 12	0.95	0	0.42	0.05
Mxa	5	− 0.06	0.77	− 0.21	0.34
PI	11	− 0.1	0.64	− 0.23	0.29
CrCP	− 9	0.8	0	0.17	0.43
DCM	− 34	0.61	0.01	0.36	0.09
ETCO_2_	− 3	0.04	0.84	− 0.01	0.95

ABP_FINGER_ (mm Hg), arterial blood pressure monitored at the auditory meatus level; FV (cm/s), cerebral blood flow velocity; nICP (mm Hg), non-invasive intracranial pressure; nCPP (mm Hg), non-invasive cerebral perfusion pressure; Mxa, cerebral autoregulation index; PI, pulsatility index; CrCP (mm Hg), critical closing pressure; DCM (mm Hg), diastolic closing margin; ETCO_2_ (kPa), end-tidal carbon dioxide concentration; ∆ (delta), variation between phases A and B in percentage; R, Spearman correlation coefficient

There was a significant difference between pre-operative and operative ABP_ARM_ [86.67 (80.83–95.67) and 76.00 (69.83–80.83), respectively; mean decrease of 9.68 mm Hg (− 12%) (*p* < 0.001)]. Between supine and BCP, significant differences occurred in DCM (mean decrease of 6.43 mm Hg (− 34%) (*p* = 0.009)) and PI (mean increase of 0.11 (+ 11%) (*p* = 0.05)). ABP_FINGER_ exhibited a decreasing trend from phase A to B, as well as nICP, nCPP, and CrCP. End tidal CO_2_ did not change significantly between the two phases.

When compared to TCD parameters in a healthy population [29], patients in this study had low FV (< 60 cm/s). PI values also deviated from normal ranges in both measurement points (PI > 0.8 [29]).

nCPP values were lower than previously reported in healthy volunteers (< 60 mm Hg [30]), and decreased further from supine to BCP. nICP presented a decreasing trend between phases; however, the observed changes in nCPP were better correlated with changes in ABP_FINGER_ than nICP [Table 3, ∆R = 0.95 (*p* < 0.001) and ∆R = 0.44 (*p* = 0.04), respectively].

Mxa showed lower levels indicating dysfunctional cerebral autoregulation (Mxa > 0.3) in phases A and B. CrCP did not approach diastolic ABP_FINGER_ from phase A to B; consequently, DCM did not reach critical values (≤ 0 mm Hg), remaining above a mean of 12 mm Hg during BCP. Changes in CrCP were strongly correlated with changes in diastolic ABP (∆R = 0.82, *p* < 0.001). Nevertheless, considering individual cases, two patients during phase B (one with history of arterial hypertension) and one patient during phase A presented relatively low values for DCM below 5 mm Hg. Generally, changes in DCM and diastolic FV were correlated [∆R = 0.40 (*p* = 0.05)].

**Fig. 1:**
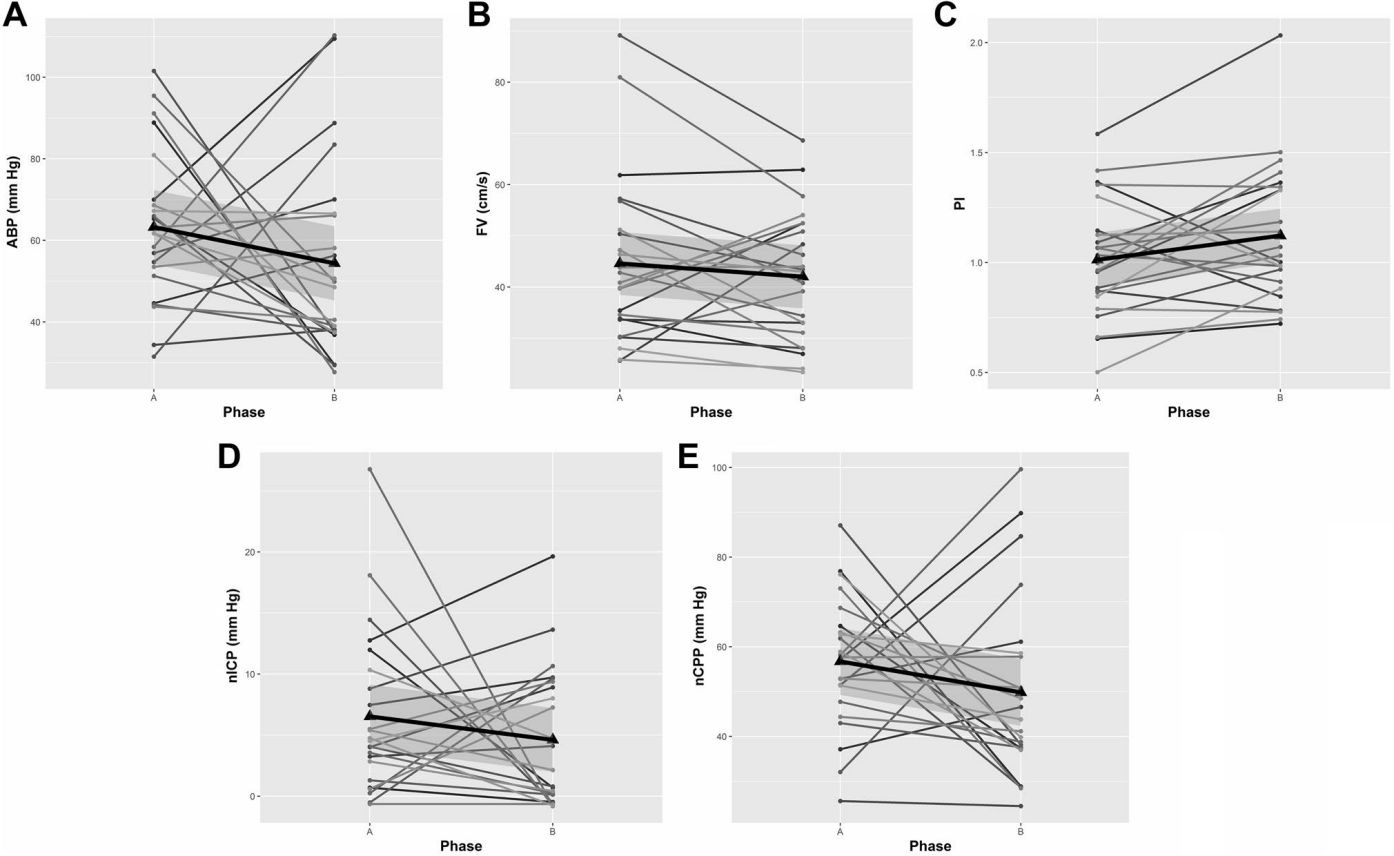
Longitudinal plots showing changes between supine (phase A) and beach chair position (phase B) for ABP_FINGER_ (**a**), FV (**b**), PI (**c**),nICP (**d**), and nCPP (**e**). Triangles on the plots represent the mean values for each variable at a specific surgical phase. Thick black lines represent the linear fit of the data; grey shadowed areas represent the 95% confidence interval of the linear model representative of the data. *ABP* arterial blood pressure, *FV* cerebral blood flow velocity, *PI* pulsatility index, *nICP* non-invasive intracranial pressure, *nCPP* non-invasive cerebral perfusion pressure

**Fig. 2:**
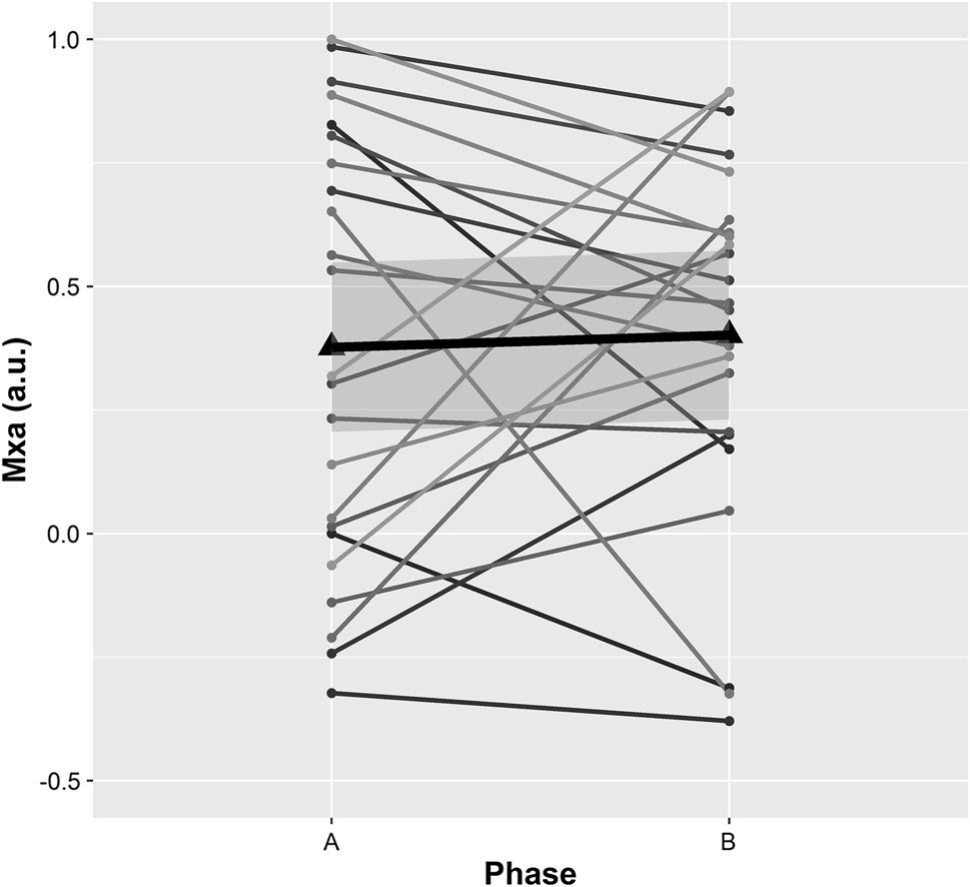
Longitudinal plot showing changes between supine (phase A) and beach chair position (phase B) for cerebral autoregulation index (Mxa). Triangles on the plots represent the mean values for each variable at a specific surgical phase. Thick black lines represents the linear fit of the data; grey shadowed areas represent the 95% confidence interval of the linear model representative of the data

**Fig. 3:**
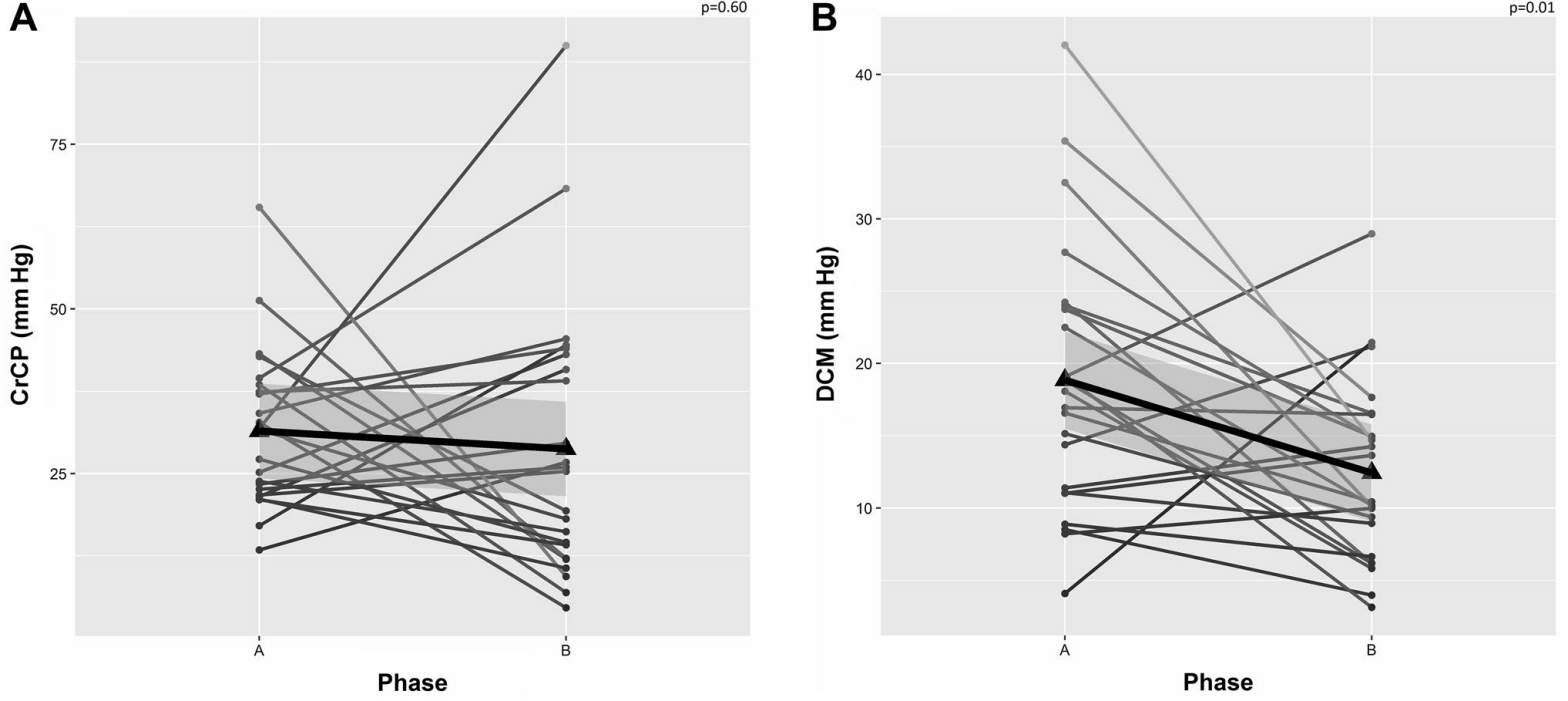
Longitudinal plots showing changes between supine (phase A) and beach chair position (phase B) for critical closing pressure (CrCP—**a**) and the diastolic closing margin (DCM—**b**). Triangles on the plots represent the mean values for each variable at a specific surgical phase. Thick black lines represent the linear fit of the data; grey shadowed areas represent the 95% confidence interval of the linear model representative of the data

No patient presented symptoms or signs suggestive of neurological damage after shoulder surgery in our cohort. Individual and mean changes across phases from supine (A) to beach chair position (B) are shown in Fig. 1 for ABP_FINGER_, FV, PI, nICP, and nCPP; Fig. 2 for autoregulation index; and Fig. 3 for CrCP and DCM.

## Discussion

Our study demonstrates that changing from the supine to beach chair position adversely affects cerebral haemodynamics with significant decrease in DCM and increase in pulsatility index, and an impairment in all cerebral haemodynamics parameters.

In previous studies reporting catastrophic perioperative brain infarction associated with shoulder surgery in the sitting position, the patients did not present any risk factors for perioperative stroke, and the authors attributed these complications to intraoperative cerebral hypoperfusion [6].

Cerebral hypoperfusion can be represented by the low cerebral blood flow velocity pattern observed in our study, which was even lower than previous findings in a study assessing normative values in healthy volunteers [29]. This may be explained by the fact that arterial blood pressure and estimated cerebral perfusion pressure were below 60 mm Hg in both surgery phases. It suggests that the patients were on the verge of the lower limit of autoregulation [31, 32], where FV decreases passively with decreasing ABP or CPP [20]. Pulsatility index, as a parameter describing changes in the TCD waveform resulting from changes in CPP, deviated from the normal range under these conditions if compared to normative values in healthy volunteers [29]. However, PI did not show high values as indicated in previous studies assessing cerebral haemodynamics in patients with traumatic brain injury, for instance (PI > 2) [21]. In our study, we observed that PI increased whereas CPP decreased, although they did not present a significant correlation (∆R = − 0.15, *p* = 0.49). Therefore, in this case, PI could be better described as an indicator of increased cerebrovascular resistance.

Diminished cerebral autoregulation has been reported in patients undergoing shoulder surgery in the beach chair position previously. A study from Laflam et al. [33], applying near-infrared spectroscopy (NIRS) for CA assessment using COx [a correlation index between ABP and regional cerebral oxygen saturation (rSCO_2_)] indicated that these patients were more likely to have impaired autoregulation and lower rSCO_2_ in comparison to patients undergoing surgery in the lateral decubitus position. Moreover, in a study from Hayashi et al. [34], decreases in rSCO_2_ were proportional to reductions in cortex level ABP (calibrated at the level of the auditory meatus) induced by postural changes due to the BCP. Although cerebral desaturation events assessed with NIRS are commonly observed during shoulder surgery in the BCP, a recent study from Cox et al. [35] revealed that patients did not have significant cognitive deficits after this procedure. To date, although other authors have applied TCD as a tool for cerebral haemodynamic assessment during BCP [33], ours is the first reporting that TCD-based CA was dysfunctional in this surgical setting.

Our findings suggest that the patients were out of the cerebral autoregulatory range in both positions. Considering that these patients did not present any previous indications of neurological impairment, the absence of a functional CA during supine position could be attributed to the effects of anaesthetic agents on arterial blood pressure, and ultimately on cerebral circulation. Propofol has been reported to preserve autoregulation either at high or low doses in healthy individuals [36]. However, high doses of this drug have been shown to impair CA in TBI patients [37]. Sevoflurane in standard doses has not been reported to alter global CBF and CA [38, 39]; however, alterations on regional CBF have been reported with inhaled anaesthetic agents [40]. Although the anaesthetic agents used in the study have not been reported to alter CA, we hypothesise that an early ABP hypotensive effect and decrease in systemic vascular resistance [41] after administration of propofol could have been the cause for a dysfunctional of CA during the supine phase, as monitoring was performed immediately after anaesthesia induction and therefore did not account for vasopressor administration to overcome the hypotension event.

Changes in estimated cerebral perfusion pressure were strongly correlated with changes in ABP monitored at the auditory meatus level (∆R = 0.82, *p* < 0.001). It is believed that the gravitational effects of BCP contribute to the observed arterial hypotension and consequent CPP decrease, as in anaesthetised patients an altered baroreflex compromises haemodynamic control in the sitting position [42]. Arterial hypotension is commonly reported in procedures performed in this surgical setting, which has been reported to produce ABP decreases of 20% in an extensive study including 5177 patients undergoing neurosurgery and orthopaedic surgeries. 50% of these patients experienced severe hypotension defined as an ABP decrease ≥ 40% relative to pre-operative values [43]. In another study including 4169 shoulder surgery procedures, 47% of patients presented hypotension defined as an ABP < 66 mm Hg or a decrease > 30% from pre-anaesthesia values. 37% of these patients experienced severe hypotension [9]. Other studies from McCulloch et al. [12], Hanouz et al. [13] and Buget et al. [44] also reported decreases in ABP after transitioning from supine to BCP.

Although the decrease in ABP_FINGER_ reached an average of 14% (also observed for ABP_ARM_—12% decrease), besides cerebral hypoperfusion, we observed that such changes from supine to BCP were also reflected in DCM [Table 2, ∆ of − 34%, delta correlation with ABP, R = 0.8 (*p* < 0.001)]. By monitoring CrCP, DCM can be trended and may represent an important clinical threshold in patients with arterial hypotension [28], as it can provide to the clinician a safety margin for manipulation of ABP in the operative setting.

Given the association of these modelled parameters with the vasomotor tone of blood vessels, CrCP and DCM logically relate to changes in previously available TCD parameters, such as the mean or diastolic flow velocity values. However, mathematical models may improve our comprehension of cerebral haemodynamics over the existing TCD parameters in a variety of conditions affecting the brain, provided that they are able to outline the main relationships among several physiological parameters in quantitative terms [45]. In critical conditions, for example, cerebral circulatory arrest, while a decreasing diastolic flow velocity towards zero only indicates the imminence of this event to the clinician, CrCP could provide information on which specific blood pressure target the clinician should avoid to prevent a circulatory arrest. In a scenario where neuromonitoring is essential to the management of patients, more comprehensive parameters like CrCP and DCM could provide precision to certain interventions and individualise the treatment.

We could not identify any cases in which DCM decreased to 0 mm Hg, although it decreased significantly between phases (Fig. 3). Nevertheless, in three isolated cases, we could observe DCM approaching relatively low values (~ 5 mm Hg), which could have been the ideal scenario for fluid and vasopressor administration to increase ABP towards safe levels concerning cerebral perfusion. In addition, overall changes in DCM and diastolic FV were correlated, indicating a decrease in FV during diastole and consequent increase in pressure passivity to cerebral circulation [28].

For instance, in a study of Hanouz et al. [13], the authors reported that beach chair position-induced ABP decrease, requiring vasopressors and fluid challenge in 44 patients (83% of the study’s cohort). In this study, the authors only relied on ABP decrease of 20% from pre-induction values to guide the vasopressors intervention, only considering mean FV for the assessment of cerebral haemodynamics.

Given our findings, continuous multiparameter TCD monitoring could provide a more comprehensive interpretation of the haemodynamic changes and guide individualised management of ABP and cerebral haemodynamics in the BCP operative setting. A special scenario would be combining ABP and multiparameter TCD monitoring for cases at imminent risk of cerebrovascular derangements, such as in patients with idiopathic intracranial hypertension, hydrocephalus, diabetes, arterial hypertension, and history of stroke. In these cases, special attention should be given to manage ABP within normal ranges with vasopressors or fluids before and throughout beach chair position. In this context, recent studies have even suggested the use of invasive arterial lines to improve the monitoring of ABP and consequently cerebral blood flow in the BCP operative setting [46, 47]. Moreover, the technique of calibrating invasive arterial blood pressure at the auditory meatus level could potentially prevent low cerebral perfusion pressure in patients positioned with an elevated head.

Furthermore, from a surgical point of view, given the observed arterial hypotension leading to dysfunctional cerebral autoregulation and other potential cerebrovascular risks, a possibility to be considered is performing the procedure with only a regional block instead of general anaesthesia. Under these conditions, the normal functioning of cardiovascular reflex responses could prevent events of arterial hypotension in the beach chair position.

This study has potential limitations. First, is the small number of patients included in the study analysis, which was inferior to the calculated sample size. Nevertheless, given the limited study period and the absence of potential eligible patients during this time, we could not accomplish the established sample size. However, we could obtain consistent results even with the reduced number of patients included in the study. Baseline FV and ABP were not monitored before anaesthesia induction. We hypothesized that supine position after induction should be the baseline phase, as the effects of anaesthesia on cerebral haemodynamics should be considered. Moreover, due to surgical configurations, we could not extend monitoring of the parameters throughout surgery. However, according to Hanouz et al. [13], significant changes in cerebral haemodynamics occurred only from baseline (after anaesthesia induction in supine position) to BCP. Because of technical reasons of limited working space in the operating room, we could only assess FV ipsilateral to the side of surgery, therefore not accounting for differences in flow that may exist between left and right MCAs. In this context, because the interscalene block was performed ipsilateral to the side of FV recordings, there is the potential for that sympathetic blockade to have altered CBF during ABP hypotension [48]. ABP_ARM_ and ABP_FINGER_ could not be compared effectively in any stages of the experimental protocol since simultaneous recordings of these parameters were unfeasible. We have presented both variables for the sake of a qualitative comparison only, to trace a parallel between changes in ABP observed clinically (ABP_ARM_) and experimentally (ABP_FINGER_). We also acknowledge the heterogeneity among patients as to individual changes in ABP and FV, however, we can only speculate its causes. For instance, the effects of anaesthesia with consequent absence of the cardiovascular reflex responses could have influenced ABP and FV across the wide age span of the patients recruited.

Short periods of FV and ABP monitoring makes the autoregulation index calculation potentially less reliable. Thirty minutes is considered as a standard, but shorter periods were also used in the past (1-min epochs over time) [49]. Increasing the monitoring time in our study setting would increase surgery time, which would result in additional stress on patients and surgical teams.

Another potential limitation is the absence of the gold standard invasive technique for ICP monitoring, which would have allowed the determination of the degree of accuracy of TCD in estimating ICP and CPP. However, invasive monitoring of ICP cannot be considered in a cohort of patients without any indications of brain injury. In TBI patients, the nICP estimation method used in this work showed a 95% confidence interval for ICP prediction of 9.94 mm Hg. Although the accuracy of the method is not ideal at the current stage of development, it has shown good prediction ability for detection of ICP increases associated with changes in cerebral blood volume (ROC area under the curve of 0.82), as well as good correlation with standard invasive methods to track changes of ICP in time non-invasively (R = 0.80) [50]. However, considering that the nICP method algorithm was derived from a TBI population, in which ICP changes are of greater magnitude in comparison to the cohort studied here, it is supposed that it would underestimate ICP in subjects presenting a normal ICP range. This inaccuracy is suggested to be associated with the matter of non-specific nICP calibrations.

In conclusion, our study suggests altered cerebral haemodynamics in patients undergoing shoulder surgery in the beach chair position. The driving force for such a  pattern was an arterial blood pressure staying out of the assumed cerebral autoregulatory range for both supine and beach chair positions. Nevertheless, the gravitational effects to which patients were subjected during BCP caused a further worsening of the cerebral hemodynamic parameters. These findings may have particular significance in patients with clinically overt or covert cerebrovascular disease, especially in an older population or patients with the latter condition in whom the outcome may be more catastrophic.

## References

[1] Peruto CM, Ciccotti MG, Cohen SB (2009). Shoulder arthroscopy positioning: lateral decubitus versus beach chair. Arthrosc J Arthrosc Relat Surg..

[2] Trentman TL, Fassett SL, Thomas JK, Noble BN, Renfree KJ, Hattrup SJ (2011). More hypotension in patients taking antihypertensives preoperatively during shoulder surgery in the beach chair position. Can J Anaesth..

[3] Buhre W, Weyland A, Buhre K, Kazmaier S, Mursch K, Schmidt M (2000). Effects of the sitting position on the distribution of blood volume in patients undergoing neurosurgical procedures. Br J Anaesth..

[4] Bhatti MT, Enneking FK (2003). Visual loss and ophthalmoplegia after shoulder surgery. Anesth Analg..

[5] Morandi X, Riffaud L, Amlashi SFA, Brassier G (2004). Extensive spinal cord infarction after posterior fossa surgery in the sitting position: case report. Neurosurgery.

[6] Pohl A, Cullen DJ (2005). Cerebral ischemia during shoulder surgery in the upright position: a case series. J Clin Anesth..

[7] Friedman DJ, Parnes NZ, Zimmer Z, Higgins LD, Warner JJP (2009). Prevalence of cerebrovascular events during shoulder surgery and association with patient position. Orthopedics.

[8] Weber SC, Abrams JS, Nottage WM (2002). Complications associated with arthroscopic shoulder surgery. Arthroscopy.

[9] Yadeau JT, Casciano M, Liu SS, Edmonds CR, Gordon M, Stanton J (2011). Stroke, regional anesthesia in the sitting position, and hypotension: a review of 4169 ambulatory surgery patients. Reg Anesth Pain Med..

[10] Papadonikolakis A, Wiesler ER, Olympio MA, Poehling GG (2008). Avoiding catastrophic complications of stroke and death related to shoulder surgery in the sitting position. Arthroscopy.

[11] Drummond JC, Lee RR, Howell JP (2012). Focal cerebral ischemia after surgery in the beach chair position: the role of a congenital variation of circle of Willis anatomy. Anesth Analg..

[12] McCulloch TJ, Liyanagama K, Petchell J (2010). Relative hypotension in the beach-chair position: effects on middle cerebral artery blood velocity. Anesth Intensive Care..

[13] Hanouz J-L, Fiant A-L, Gérard J-L (2016). Middle cerebral artery blood flow velocity during beach chair position for shoulder surgery under general anesthesia. J Clin Anesth..

[14] Schmidt B, Klingelhöfer J, Schwarze JJ, Sander D, Wittich I (1997). Noninvasive prediction of intracranial pressure curves using transcranial doppler ultrasonography and blood pressure curves. Stroke.

[15] Kasuga Y, Nagai H, Hasegawa Y, Nitta M (1987). Transmission characteristics of pulse waves in the intracranial cavity of dogs. J Neurosurg..

[16] Marmarelis PMV (1978). Analysis of physiological systems.

[17] Cardim D, Robba C, Donnelly J, Bohdanowicz M, Schmidt B, Damian M (2015). Prospective study on non-invasive assessment of ICP in head injured patients: comparison of four methods. J Neurotrauma.

[18] Czosnyka M, Brady K, Reinhard M, Smielewski P, Steiner LA (2009). Monitoring of cerebrovascular autoregulation: facts, myths, and missing links. Neurocrit Care..

[19] Czosnyka M, Smielewski P, Kirkpatrick P, Menon DK, Pickard JD (1996). Monitoring of cerebral autoregulation in head-injured patients. Stroke.

[20] Czosnyka M, Smielewski P, Piechnik S, Steiner L, Pickard JD (2001). Cerebral autoregulation following head injury. J Neurosurg..

[21] De Riva N, Budohoski KP, Smielewski P, Kasprowicz M, Zweifel C, Steiner LA (2012). Transcranial doppler pulsatility index: What it is and what it isn’t. Neurocrit Care..

[22] Nichol J, Girling F, Jerrard W, Claxton EB, Burton AC (1951). Fundamental instability of the small blood vessels and critical closing pressures in vascular beds. Am J Physiol..

[23] Dewey RC, Pieper HP, Hunt WE (1974). Experimental cerebral hemodynamics. Vasomotor tone, critical closing pressure, and vascular bed resistance. J Neurosurg..

[24] Czosnyka M, Richards H, Pickard JD, Harris N, Iyer V (1994). Frequency-dependent properties of cerebral blood transport—an experimental study in anaesthetized rabbits. Ultrasound Med Biol..

[25] Michel E, Hillebrand S, vonTwickel J, Zernikow B, Jorch G (1997). Frequency dependence of cerebrovascular impedance in preterm neonates: a different view on critical closing pressure. J Cereb Blood Flow Metab..

[26] Puppo C, Camacho J, Yelicich B, Moraes L, Biestro A, Gomez H (2012). Bedside study of cerebral critical closing pressure in patients with severe traumatic brain injury: a transcranial Doppler study. Acta Neurochir Suppl..

[27] Varsos GV, Richards H, Kasprowicz M, Budohoski KP, Brady KM, Reinhard M (2013). Critical closing pressure determined with a model of cerebrovascular impedance. J Cereb Blood Flow Metab..

[28] Varsos GV, Richards HK, Kasprowicz M, Reinhard M, Smielewski P, Brady KM (2014). Cessation of diastolic cerebral blood flow velocity: the role of critical closing pressure. Neurocrit Care..

[29] Tegeler CH, Crutchfield K, Katsnelson M, Kim J, Tang R, Passmore Griffin L (2013). Transcranial doppler velocities in a large, healthy population. J Neuroimaging..

[30] Bratton SL, Chestnut RM, Ghajar J, McConnell Hammond FF, Harris OA, Hartl R (2007). Guidelines for the management of severe traumatic brain injury. IX. Cerebral perfusion thresholds. J Neurotrauma..

[31] Lassen NA, Christensen MS (1976). Physiology of cerebral blood flow. Br J Anaesth..

[32] Paulson OB, Strandgaard S, Edvinsson L (1990). Cerebral autoregulation. Cerebrovasc Brain Metab Rev..

[33] Laflam A, Joshi B, Brady K, Yenokyan G, Brown C, Everett A (2015). Shoulder surgery in the beach chair position is associated with diminished cerebral autoregulation but no differences in postoperative cognition or brain injury biomarker levels compared with supine positioning. Anesth Analg..

[34] Hayashi K, Tanabe K, Minami K, Sakata K, Nagase K, Iida H (2017). Effect of blood pressure elevation on cerebral oxygen desaturation in the beach chair position. Asian J Anesthesiol..

[35] Cox RM, Jamgochian GC, Nicholson K, Wong JC, Namdari S, Abboud JA (2018). The effectiveness of cerebral oxygenation monitoring during arthroscopic shoulder surgery in the beach chair position: a randomized blinded study. J Shoulder Elb Surg..

[36] Strebel S, Lam AM, Matta B, Mayberg TS, Aaslid R, Newell DW (1995). Dynamic and static cerebral autoregulation during isoflurane, desflurane, and propofol anesthesia. Anesthesiology..

[37] Grathwohl KW, Black IH, Spinella PC, Sweeney J, Robalino J, Helminiak J (2008). Total intravenous anesthesia including ketamine versus volatile gas anesthesia for combat-related operative traumatic brain injury. Anesthesiology.

[38] Summors AC, Gupta AK, Matta BF (1999). Dynamic cerebral autoregulation during sevoflurane anesthesia: a comparison with isoflurane. Anesth Analg..

[39] Gupta S, Heath K, Matta BF (1997). Effect of incremental doses of sevoflurane on cerebral pressure autoregulation in humans. Br J Anaesth..

[40] Schlünzen L, Cold GE, Rasmussen M, Vafaee MS (2006). Effects of dose-dependent levels of isoflurane on cerebral blood flow in healthy subjects studied using positron emission tomography. Acta Anaesthesiol Scand..

[41] Hug CC, McLeskey CH, Nahrwold ML, Roizen MF, Stanley TH, Thisted RA (1993). Hemodynamic effects of propofol: Data from over 25,000 patients. Anesth Analg..

[42] Smith JJ, Porth CM, Erickson M (1994). Hemodynamic response to the upright posture. J Clin Pharmacol..

[43] Pin-On P, Schroeder D, Munis J (2013). The hemodynamic management of 5177 neurosurgical and orthopedic patients who underwent surgery in the sitting or “beach chair” Position without incidence of adverse neurologic events. Anesth Analg..

[44] Buget MI, Atalar AC, Edipoglu IS, Sungur Z, Sivrikoz N, Karadeniz M (2016). Patient State Index e alterações do fluxo sanguíneo cerebral durante artroscopia do ombro em posição de cadeira de praia. Braz J Anesthesiol..

[45] Ursino M, Lodi CA (1997). A simple mathematical model of the interaction between intracranial pressure and cerebral hemodynamics. J Appl Physiol..

[46] Gabriel RA, Beverly A, Dutton RP, Urman RD (2017). Patterns of intra-arterial blood pressure monitoring for patients undergoing total shoulder arthroplasty under general anesthesia: a retrospective analysis of 23,073 patients. J Clin Monit Comput..

[47] Luedi MM, Bendjelid K (2017). Hemodynamic monitoring during surgeries in beach chair position: what can a big picture teach us?. J Clin Monit Comput..

[48] Visocchi M, Chiappini F, Cioni B, Meglio M (1996). Cerebral blood flow velocities and trigeminal ganglion stimulation. A transcranial Doppler study. Stereotact Funct Neurosurg..

[49] Reinhard M, Roth M, Müller T, Czosnyka M, Timmer J, Hetzel A (2003). Cerebral autoregulation in carotid artery occlusive disease assessed from spontaneous blood pressure fluctuations by the correlation coefficient index. Stroke.

[50] Cardim D, Schmidt B, Robba C, Donnelly J, Puppo C, Czosnyka M (2016). Transcranial doppler monitoring of intracranial pressure plateau waves. Neurocrit Care..

